# Supplementary material for: Red muscle activity in bluegill sunfish Lepomis macrochirus during forward accelerations
Source: Sci Rep. 2019 May 30;9:8088. doi: 10.1038/s41598-019-44409-7 (PMC6542830; doi:10.1038/s41598-019-44409-7)
Supplement: Supplementary file 1 — Supplementary information [file 41598_2019_44409_MOESM1_ESM.docx]

Supplementary Information

**Red muscle activity in bluegill sunfish *Lepomis macrochirus* during forward accelerations**

Margot A. B. Schwalbe, Alexandra L. Boden, Tyler N. Wise, and Eric. D. Tytell

**Table S1.** Results from two-way mixed regression models with autoregressive covariance structure (AR1) that includes fixed, random, and repeated effects for kinematic data.

| **Source/Effect*** | **F** | **df** | **p** | **Individual**  **variance** | **Autoregressive covariance** |
| --- | --- | --- | --- | --- | --- |
| Tail beat frequency (log) |  |  |  | 0.061 | 0.77 |
| **Acceleration** | **125.2** | **3,3303** | **<0.0001** |  |  |
| **Swimming speed** | **4.8** | **4,172** | **0.0011** |  |  |
| Acceleration × Speed | 2.1 | 12,3303 | 0.0137 |  |  |
| Wave speed (log) |  |  |  | 0.056 | 0.65 |
| **Acceleration** | **140.3** | **3,3303** | **<0.0001** |  |  |
| Swimming speed | 3.1 | 4,172 | 0.0162 |  |  |
| **Acceleration × Speed** | **2.9** | **12,3303** | **0.0005** |  |  |
| Head amplitude |  |  |  | 0.010 | 0.61 |
| **Acceleration** | **12.0** | **3,3303** | **<0.0001** |  |  |
| Swimming speed | 3.4 | 4,172 | 0.0112 |  |  |
| Acceleration × Speed | 1.9 | 12,3303 | 0.0321 |  |  |
| Tail amplitude |  |  |  | 0.019 | 0.69 |
| **Acceleration** | **83.5** | **3,3303** | **<0.0001** |  |  |
| Swimming speed | 1.7 | 4,172 | 0.2165 |  |  |
| Acceleration × Speed | 1.5 | 12,3303 | 0.1246 |  |  |
| Body wavelength |  |  |  | 0.055 | -0.02 |
| **Acceleration** | **7.9** | **3,3303** | **<0.0001** |  |  |
| **Swimming speed** | **12.6** | **4,172** | **<0.0001** |  |  |
| Acceleration × Speed | 1.4 | 12,3303 | 0.1779 |  |  |

*N = 3,499 for all models

**Table S2.** Results from two-way multivariate ANOVA tests and principal components analysis on kinematic data.

| **Source/Effect*** | **F** | **df** | **p** |
| --- | --- | --- | --- |
| Multivariate |  |  |  |
| **Acceleration** | **270.8** | **9^†^** | **<0.0001** |
| **Swimming speed** | **52.7** | **12^†^** | **<0.0001** |
| **Acceleration × Speed** | **8.2** | **36^†^** | **<0.0001** |
| Univariate PC1 (47.5% of variance) | |  |  |
| **Acceleration** | **1472.5** | **3^‡^** | **<0.0001** |
| **Swimming speed** | **38.7** | **4^‡^** | **<0.0001** |
| **Acceleration × Speed** | **7.9** | **12^‡^** | **<0.0001** |
| Univariate PC2 (24.8% of variance) | |  |  |
| Acceleration | 1.3 | 3^‡^ | 0.2766 |
| **Swimming speed** | **109.9** | **4^‡^** | **<0.0001** |
| **Acceleration × Speed** | **7.9** | **12^‡^** | **<0.0001** |
| Univariate PC3 (18.1% of variance) | |  |  |
| **Acceleration** | **8.0** | **3^‡^** | **<0.0001** |
| **Swimming speed** | **13.6** | **4^‡^** | **<0.0001** |
| **Acceleration × Speed** | **8.5** | **12^‡^** | **<0.0001** |

*N = 3,499 for all models; ^†^denominator degrees of freedom = 10,437; ^‡^denominator degrees of freedom = 3,479

**Table S3.** Results from three-way mixed regression models with autoregressive covariance structure (AR1) that includes fixed, random, and repeated effects for EMG data.

| **Source/Effect** | **F** | **df** | **p** | **Individual**  **variance** | **Autoregressive covariance** |
| --- | --- | --- | --- | --- | --- |
| Burst duration (N = 9,289) |  |  |  | 15.1 ms | 0.41 |
| **Acceleration** | **41.4** | **3,8258** | **<0.0001** |  |  |
| **Swimming speed** | **6.1** | **4,172** | **0.0001** |  |  |
| **EMG position** | **32.9** | **3,775** | **<0.0001** |  |  |
| **Acceleration × Speed** | **3.6** | **12,8258** | **<0.0001** |  |  |
| Acceleration × EMG position | 0.7 | 9,8258 | 0.7289 |  |  |
| Speed × EMG position | 0.8 | 12,775 | 0.6231 |  |  |
| Acceleration × EMG × Speed | 1.5 | 36,8258 | 0.0193 |  |  |
| Burst duty cycle (N = 9,302) |  |  |  | 0.041 | 0.17 |
| **Acceleration** | **99.8** | **3,8271** | **<0.0001** |  |  |
| Swimming speed | 1.1 | 4,172 | 0.3509 |  |  |
| **EMG position** | **156.4** | **3,775** | **<0.0001** |  |  |
| Acceleration × Speed | 0.8 | 12,8271 | 0.6071 |  |  |
| **Acceleration × EMG position** | **3.1** | **9,8271** | **0.0011** |  |  |
| **Speed × EMG position** | **3.0** | **12,775** | **0.0004** |  |  |
| **Acceleration × EMG × Speed** | **2.1** | **36,8271** | **0.0001** |  |  |
| Burst overlap (N = 5,023) |  |  |  | 8.8 ms | 0.43 |
| **Acceleration** | **255.4** | **3,4381** | **<0.0001** |  |  |
| Swimming speed | 2.3 | 4,165 | 0.0576 |  |  |
| **EMG position** | **23.4** | **3,429** | **<0.0001** |  |  |
| **Acceleration × Speed** | **3.2** | **12,4381** | **0.0001** |  |  |
| Acceleration × EMG position | 0.9 | 9,4381 | 0.5128 |  |  |
| **Speed × EMG position** | **2.3** | **12,429** | **0.0073** |  |  |
| Acceleration × EMG × Speed |  |  | * |  |  |
| Strain rate during active shortening (log) (N = 6,699) |  |  |  | 0.090 | 0.51 |
| **Acceleration** | **230.9** | **3,5944** | **<0.0001** |  |  |
| **Swimming speed** | **6.2** | **4,130** | **0.0001** |  |  |
| **EMG position** | **177.2** | **3,578** | **<0.0001** |  |  |
| **Acceleration × Speed** | **6.2** | **12,5944** | **<0.0001** |  |  |
| Acceleration × EMG position | 2.2 | 9,5944 | 0.0218 |  |  |
| Speed × EMG position | 1.6 | 12,578 | 0.0841 |  |  |
| Acceleration × EMG × Speed |  |  | * |  |  |
| Strain rate during active lengthening (log) (N = 6,603) |  |  |  | 0.109 | 0.48 |
| **Acceleration** | **137.2** | **3,5845** | **<0.0001** |  |  |
| **Swimming speed** | **4.4** | **4,129** | **0.0024** |  |  |
| **EMG position** | **335.0** | **3,582** | **<0.0001** |  |  |
| **Acceleration × Speed** | **2.6** | **12,5845** | **0.0016** |  |  |
| **Acceleration × EMG position** | **7.5** | **9,5845** | **<0.0001** |  |  |
| **Speed × EMG position** | **3.5** | **12,582** | **0.0001** |  |  |
| Acceleration × EMG × Speed |  |  | * |  |  |

* Not possible to estimate because of missing values.

**Table S4.** Results from the circular ANOVA test (Harrison-Kanji).

|  | **Burst onset** | | |  | **Burst offset^†^** | | |
| --- | --- | --- | --- | --- | --- | --- | --- |
| **Source^*^** | **F** | **df** | **p** |  | **χ^2^** | **df** | **p** |
| Acceleration | **302.9** | **3, 9272** | **<0.0001** |  | 13.8 | 6 | 0.0319 |
| EMG position | **24.19.8** | **3, 9272** | **<0.0001** |  | **9083.1** | **6** | **<0.0001** |
| Acceleration × EMG position | **46.1** | **9, 9272** | **<0.0001** |  | **104.2** | **9** | **<0.0001** |

* N = 9,288 for both tests. †Using *χ*^2^ test statistic because circular dispersion $\kappa>0.2$


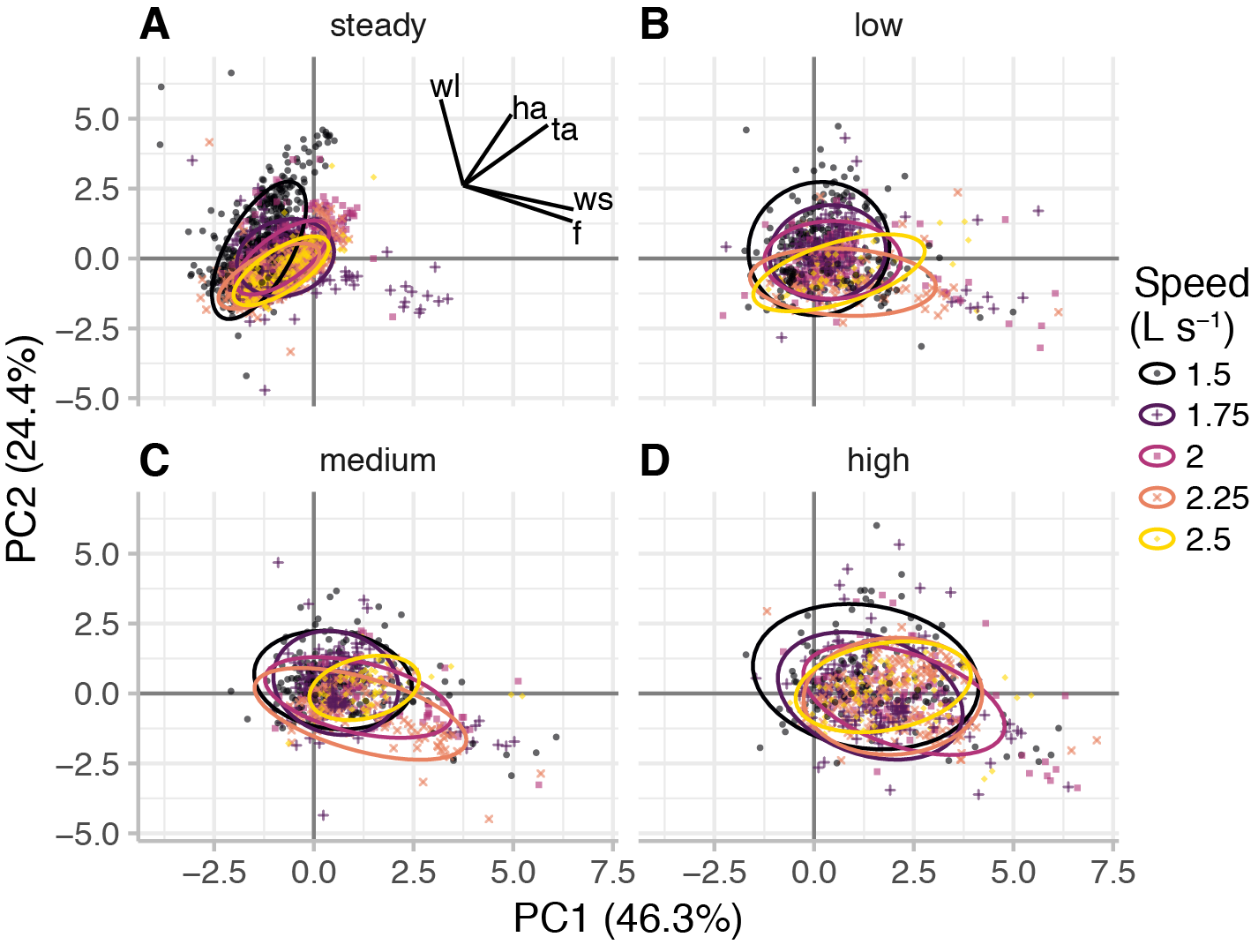


**Figure S1.** Swimming kinematics during accelerations are significantly different from those during steady swimming. (A) Steady swimming. (B) Low acceleration. (C) Medium acceleration. (D) High acceleration. The plots show principal components 1 and 2 for the acceleration groups and swimming speeds, with 95% confidence ellipses around each speed at each acceleration. Color shows speed. Tail amplitude, body wave speed, and tail beat frequency contribute most of the variation in principal component 1 (PC1), while body wavelength and head and tail amplitudes contribute most to component 2 (PC2). (ws = body wave speed, ha = head amplitude, ta = tail amplitude, f = tail beat frequency, wl = body wavelength).

**Movie Legends**

**Movie S1.** Bluegill sunfish (total length, TL = 16.5 cm) steady swimming at 2.0 L s^-1^. View from the ventrally positioned camera played back at 6% real time and the vertical dashed line indicates the video’s synchronization with forward acceleration (white), tail position (white, dashed), left and right pairs of EMG2 (red) and EMG3 (green), roll (dark blue, dashed), pitch (blue), and yaw (brown, dashed).

**Movie S2.** Bluegill sunfish (TL = 15.0 cm) accelerating beyond 1.5 L s^-1^. View from the ventrally positioned camera played back at 6% real time and the vertical dashed line indicates the video’s synchronization with forward acceleration (white), tail position (white, dashed), left and right pairs of EMG2 (red) and EMG3 (green), roll (dark blue, dashed), pitch (blue), and yaw (brown, dashed).
